# Developmental Stage Determines the Accumulation Pattern of UV-Absorbing Compounds in the Model Liverwort *Marchantia polymorpha* subsp. *ruderalis* under Controlled Conditions

**DOI:** 10.3390/plants10030473

**Published:** 2021-03-03

**Authors:** Gonzalo Soriano, María-Ángeles Del-Castillo-Alonso, Laura Monforte, Rafael Tomás-Las-Heras, Javier Martínez-Abaigar, Encarnación Núñez-Olivera

**Affiliations:** 1 Facultad de Ciencia y Tecnología, Universidad de La Rioja, Madre de Dios 53, 26006 Logroño, Spain; gonzalo.soriano@unirioja.es (G.S.); maria-angeles-del.castillo@unirioja.es (M.-Á.D.-C.-A.); laura.monforte@unirioja.es (L.M.); rafael.tomas@unirioja.es (R.T.-L.-H.); encarnacion.nunez@unirioja.es (E.N.-O.); 2 Departamento de Genética Molecular de Plantas, Centro Nacional de Biotecnología (CNB), Consejo Superior de Investigaciones Científicas (CSIC), Darwin 3, 28049 Madrid, Spain

**Keywords:** bryophytes, liverworts, *Marchantia polymorpha* subsp. *ruderalis*, ultraviolet radiation, developmental stage, phenolic compounds, apigenin, luteolin, flavone, water content

## Abstract

The liverwort *Marchantia polymorpha* subsp. *ruderalis* is an emerging model plant, and some data are available on its responses to ultraviolet (UV) radiation. However, it is unknown if the developmental stage of the thalli modulates the effects of UV radiation on the contents of potentially protecting phenolic compounds. To fill this gap, liverwort samples were exposed or non-exposed to UV radiation for 38 days under controlled conditions, using three developmental stages: gemmae (G), one-month thalli (T1), and two-month thalli (T2). Then, the bulk level of methanol-soluble UV-absorbing compounds and the contents of six flavones (apigenin and luteolin derivatives) were measured. The UV responsiveness decreased with thallus age: G and T1 plants were the most UV-responsive and showed a strong increase in all the variables, with G plants more responsive than T1 plants. In UV-exposed T2 plants, only apigenin derivatives increased and more modestly, probably due to a lower acclimation capacity. Nevertheless, the thalli became progressively tougher due to a decreasing water content, representing a possible structural protection against UV. In UV-exposed plants, the temporal patterns of the accumulation of phenolic compounds were compound-specific. Most compounds decreased with thallus age, but di-glucuronide derivatives showed a bell-shaped pattern, with T1 plants showing the highest contents. A Principal Components Analysis (PCA) ordination of the different samples summarized the results found. The patterns described above should be taken into account to select thalli of an adequate developmental stage for experiments investigating the induction of phenolic compounds by UV radiation.

## 1. Introduction

The biochemical and physiological processes occurring in plants, and their responses to the environment, are affected by the developmental stage of tissues and organs [1], which is usually related to their age. In bryophytes, physiological variations between young and mature (or old) shoots and thalli have been found. Compared to old shoots, young shoots usually show a higher physiological activity, normally linked to higher photosynthesis and respiration rates, higher chlorophyll concentrations, and higher values of the maximum quantum yield of photosystem II [2,3,4]. In addition, differences in nutrients and heavy metals along the bryophyte profile have been studied in detail [5,6,7,8], as well as the distribution of waxes and other lipids as a function of shoot age [9,10]. However, the distribution of phenolic compounds in the bryophyte gametophore, as influenced by the developmental stage, has received less attention [4], despite its importance as a protection mechanism against various adverse factors, such as cold, desiccation, herbivory or ultraviolet (UV) radiation. In particular, no study has been conducted to our knowledge on the effects of UV radiation on the accumulation of phenolic compounds in different developmental stages of bryophytes.

In tracheophytes, several studies have compared the phenolic contents in leaves of different ages, or along the development of a specific leaf, as influenced by UV radiation. In this regard, the results obtained have been diverse. Young leaves may accumulate higher amounts of phenolic compounds than old leaves when exposed to UV radiation [11,12,13,14], although in some cases flavonoid accumulation can be induced by UV in old leaves [15,16,17]. This diversity of results can be due, among other factors, to the species and the compound considered.

UV radiation is an important environmental factor influencing photosynthetic organisms [18]. Solar UV is divided into UV-C (100–280 nm), UV-B (280–315 nm) and UV-A (315–400 nm) radiation, of which only UV-A and UV-B (at wavelengths greater than 290 nm) reach the biosphere. The UV fraction represents around 5% of the solar radiation and it is mostly composed (95%) of UV-A, whereas UV-B constitutes the remaining 5%. Traditionally, UV radiation has been considered to be a harmful factor for plants, due to the diverse physiological damage that a UV excess can produce [18]. However, UV radiation is currently considered to be a general regulator inducing a number of acclimation responses in the plant.

The liverwort *Marchantia polymorpha* subsp. *ruderalis* is an emerging model species in plant research, particularly in molecular biology, on the basis of several favorable features: a haploid-dominating life cycle, which facilitates genetic analyses; its easy cultivation and cloning, starting from asexual propagules (gemmae); its usefulness in forward and reverse genetics, genetic transformation and genome-wide studies; and the general phylogenetic importance of liverworts [19]. The purpose of using this species is multifaceted because it allows a better understanding of different aspects of the physiology, molecular biology, development, and evolution of plants [19]. Given the relevance of this liverwort as a model species, it is important to know the basic aspects of its physiology and responses to environmental factors, including UV radiation. Several studies have been performed in this respect [20,21,22,23,24,25,26,27,28], but the influence of the developmental stage of the thalli on the responses to UV radiation has not previously been investigated. Responses of bryophytes to UV radiation are evolutionarily important, given that these plants were crucial in the embryophyte colonization of land [18,24], where they had to cope with multiple stressors, among them high levels of UV radiation.

In this context, our aim was to study the interaction between UV radiation and the developmental stage of the thalli on the accumulation patterns of phenolic UV-absorbing compounds in the model liverwort *M. polymorpha* subsp. *ruderalis* under controlled conditions. Specifically, we wanted to test if young thalli were more UV-reactive than more mature thalli. If the developmental stage influenced the accumulation patterns of phenolic compounds in UV-exposed bryophytes, this fact should be taken into account to select shoots or thalli of an adequate stage for experiments investigating the induction of phenolic compounds. This would assure that an effective accumulation would take place.

## 2. Results

Samples of *Marchantia polymorpha* subsp. *ruderalis* (Tak-1 accession) of three developmental stages (G, gemmae; T1, one-month thalli; and T2, two-month thalli) were exposed to two radiation regimes (P, only photosynthetically active radiation, PAR; PAB, PAR + UV-A + UV-B) for 38 days. At the end of the exposure, the contents of water (WC), methanol-soluble UV-absorbing compounds (SUVACs), and six apigenin and luteolin derivatives were measured (Table S1).

### 2.1. Individual UV-Absorbing Compounds Found

**Figure 1:**
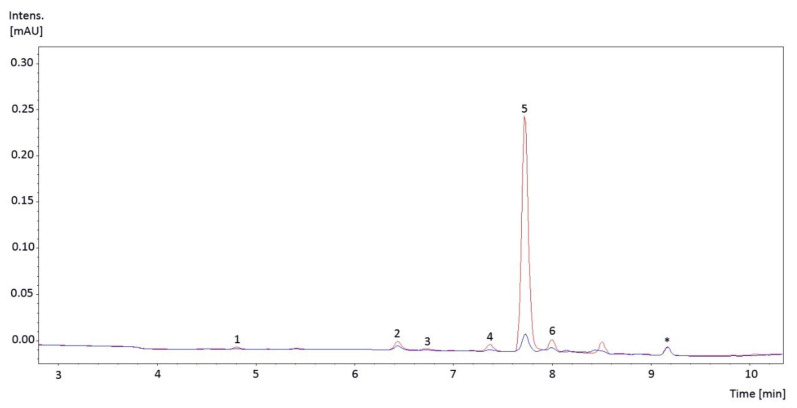
Representative ultra-performance liquid chromatography (UPLC) chromatograms of *Marchantia polymorpha* subsp. *ruderalis* Tak-1 samples exposed to the two radiation regimes imposed in the experiment: P (only only photosynthetically active radiation (PAR), blue line) and PAB (PAR + UV-A + UV-B, red line). The identified methanol-soluble UV-absorbing compounds (SUVACs) were: 1, apigenin 7,4′-di-*O*-glucuronide; 2, luteolin 7,3′-di-*O*-glucuronide; 3, luteolin 7-*O*-glucuronide; 4, luteolin 4′-*O*-glucuronide; 5, apigenin 7-*O*-glucuronide; and 6 luteolin 3′-*O*-glucuronide. Internal standard is marked with an asterisk (*).

A total of six individual SUVACs were identified in the liverwort extracts (Figure 1): apigenin 7-*O*-glucuronide, apigenin 7,4′-di-*O*-glucuronide, luteolin 3′-*O*-glucuronide, luteolin 4′-*O*-glucuronide, luteolin 7-O-glucuronide, and luteolin 7,3′-di-*O*-glucuronide. Apigenin 7-*O*-glucuronide was the most abundant compound, particularly under the PAB regime.

### 2.2. Influence of the Radiation Regime and Developmental Stage on the Physiological Variables

**Figure 2:**
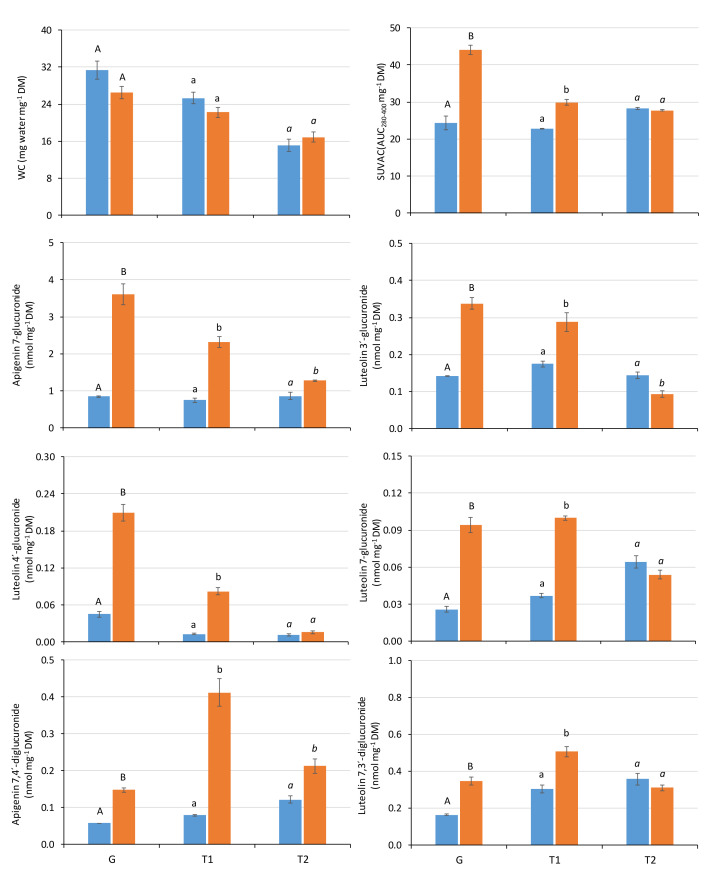
Water content (WC) values, bulk levels of soluble UV-absorbing compounds (SUVAC, in terms of the area under the absorbance curve in the interval 280–400 nm (AUC_280–400_) per DM unit), and contents of individual soluble UV-absorbing compounds in *Marchantia polymorpha* subsp. *ruderalis* Tak-1 samples, measured after 38 days of exposure to two different radiation regimes: P (only PAR, blue bars) and PAB (PAR + UV-A + UV-B, orange bars). Samples were exposed to either P or PAB regimes starting from three different developmental stages: gemmae (G), one-month thalli (T1), and two-month thalli (T2). For each developmental stage, different letters mean significant differences (at least *p* < 0.05) between radiation regimes (Student’s *t*).

A significant global effect of the radiation regime was found on every variable measured, except WC (Table 1). In addition, a significant global effect of the developmental stage was found on every variable except luteolin 7-*O*-glucuronide. The interaction between both factors was significant for every variable except WC. Thus, the effect of the radiation regime on the phenolic composition of the liverwort was modulated by the developmental stage of the thallus. Most variables showed significant differences between the three developmental stages (Table 1, Figure 2). WC, the bulk levels of SUVACs, and most mono-glucuronide derivatives (including the major compound) showed the highest values in the plants that had been exposed to UV starting from gemmae (G plants), and values decreased with the thallus age at which the samples started to be exposed (T1 and T2). In WC, this pattern occurred in both P and PAB samples. However, the bulk levels of SUVACs and most mono-glucuronide derivatives showed this pattern only in PAB samples, whereas in P samples these variables could increase (the bulk levels of SUVACs, luteolin 7-*O*-glucuronide), decrease (luteolin 4′-*O*-glucuronide) or remain more or less stable (apigenin 7-*O*-glucuronide, and luteolin 3′-*O*-glucuronide) as thallus age increased. Regarding the di-glucuronide derivatives, the highest values in PAB plants were found in the T1 stage, whereas G and T2 samples showed lower values. In P plants, values showed a slight increasing trend as thallus age increased.

The specific effect of the radiation regime on each variable and developmental stage is defined in Figure 2. WC showed no significant difference between P and PAB samples for any developmental stage. For the remaining variables, the influence of the radiation regime strongly depended on the developmental stage of the thallus. In G and T1 plants, the bulk level of SUVACs and the contents of all the individual compounds showed significantly higher values in PAB than in P samples. The magnitude of this difference was higher in G than in T1 plants for the bulk level of SUVACs and most mono-glucuronide derivatives, but not for the di-glucuronide derivatives. In T2 plants, only the contents of apigenin derivatives were higher in PAB with respect to P samples, whereas the bulk level of SUVACs and luteolin derivatives did not show any difference between regimes (except luteolin 3′-*O*-glucuronide, which decreased in PAB samples).

### 2.3. Principal Components Analysis (PCA) Ordination of Samples

**Figure 3:**
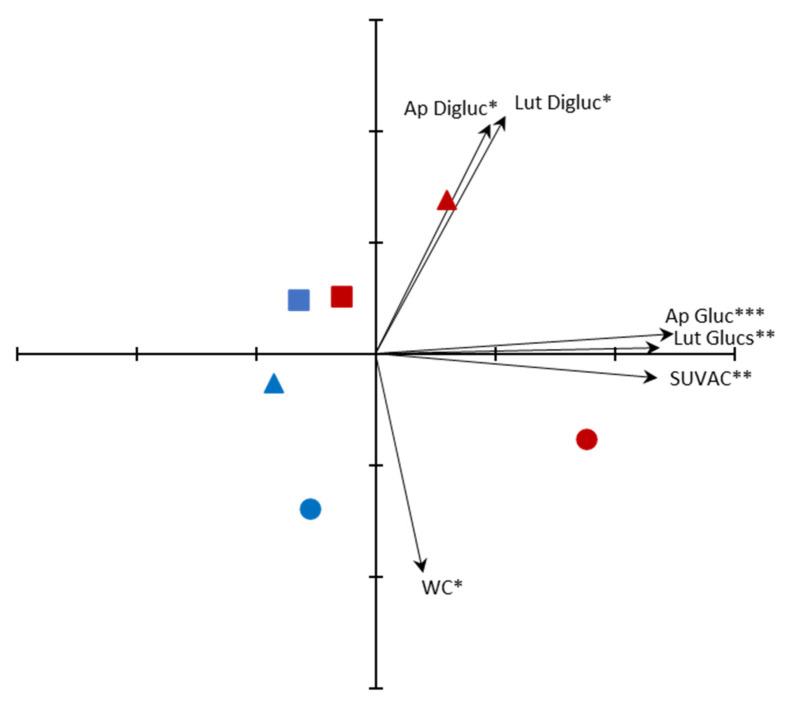
Ordination, through Principal Components Analysis (PCA), of *Marchantia polymorpha* subsp. *ruderalis* Tak-1 samples exposed to two different radiation regimes (P, only PAR, blue; PAB, PAR + UV-A + UV-B, red) starting from three different developmental stages: gemmae (G, circles), one-month thalli (T1, triangles), and two-month thalli (T2, squares). Significant loading factors for the positive and negative parts of each principal component (PC), together with their corresponding significance levels, are shown as arrows. Significance of each loading factor is denoted by asterisks (***, *p* < 0.001; **, *p* < 0.01; *, *p* < 0.05). PC1 is the horizontal one, and PC2 is the vertical one. Each tic-mark on the PCs represents one unit. Ap Digluc, apigenin 7,4′-di-*O*-glucuronide. Ap Gluc, apigenin 7-*O*-glucuronide. Lut Digluc, luteolin 7,3′-di-*O*-glucuronide. Lut Glucs, sum of luteolin 3′-*O*-glucuronide, luteolin 4′-*O*-glucuronide and luteolin 7-*O*-glucuronide. SUVAC, bulk level of methanol-soluble UV-absorbing compounds. WC, water content.

Samples of the three different developmental stages that were exposed to the two radiation regimes were ordinated by Principal Components Analysis (PCA) using the variables studied. The accumulated variance by the first two principal components (PCs) was 86.4% (56.7% for PC1 and 29.7% for PC2). The plot using these PCs, together with the loading factors and their respective significances, is shown in Figure 3. The content of the major compound (apigenin 7-*O*-glucuronide), the bulk level of SUVACs, and the sum of luteolin mono-glucuronides (luteolin 3′-*O*-glucuronide, luteolin 4′-*O*-glucuronide and luteolin 7-*O*-glucuronide) were significant loading factors for the positive part of PC1, whereas there was no significant loading factor for the negative part. The significant loading factors for the positive part of PC2 were the contents of both apigenin- and luteolin-diglucuronide derivatives, whereas WC was the only significant loading factor for its negative part. PC1 separated the samples as a function of the radiation regime to which they were exposed. P samples were located towards the negative part of the PC1, whereas most PAB samples (particularly G plants, but also T1 plants) were displaced to the positive part, on the basis of their higher bulk levels of SUVACs and higher contents of mono-glucuronide derivatives. G plants of P and PAB regimes were notably distant each other, as were T1 plants of P and PAB regimes, whereas T2 plants of P and PAB regimes were much closer. In addition, PAB plants were clearly separated along PC1 as a function of their developmental stage, whereas P plants were not. PC2 clearly ordinated P samples as a function of their developmental stage, with plants of increasing age progressively more displaced towards the positive part of the PC2, given that di-glucuronide derivative contents increased and WC decreased with age. However, this ordination disappeared in PAB plants, given that, in this regime, T1 plants showed the highest contents of di-glucuronide derivatives.

## 3. Discussion

### 3.1. Phenolic Composition of the Liverwort Samples

Six individual SUVACs (Table 1, Figure 1) were identified in the thalli of *Marchantia polymorpha* subsp. *ruderalis* (Tak-1 accession). All of them were flavones, specifically apigenin and luteolin glucuronides, whose predominance is a feature of marchantialean liverworts [29]. Flavones are one of the biggest families of flavonoids and their functions in plants include protection against UV radiation and oxidative stress, signaling, and interactions with other organisms [30,31]. In addition, both apigenins and luteolins are interesting medicinal compounds [30,32].

**Table 2:** Literature references where flavones of the three currently recognized subspecies of *Marchantia polymorpha* L. (subsp. *polymorpha*, subsp. *montivagans* Bischl. & Boissel.-Dub., and subsp. *ruderalis* Bischl. and Boissel.-Dub.) were found, differentiating the three investigated accessions of *M. polymorpha* subsp. *ruderalis* (Tak-1, a local Spanish wild accession, and Sey-1 and Aud-2 accessions). In some cases, the samples analyzed were not assigned to any subspecies or accession. To facilitate comparison with previous literature, old synonyms of *M. polymorpha* subspecies are provided ^1^.

	No Subspecies Assigned	Subsp. *polymorpha*	Subsp. *montivagans*	Subsp. *ruderalis*			
				Tak-1 Accession	Local Wild Accession	Sey-1 and Aud-2 Accessions	No Accession Assigned
Apigenin	[20,33]	[34]	-	[23,26]	[28]	-	[34]
Apigenin 7-*O*-glucoside	[33]	-	-	-	-	-	-
Apigenin 7-*O*-glucuronide	[20]	[27,29,34]	[29]	[26] (this study)	[28]	[21,22]	[29,34]
Apigenin 7,4′-di-*O*-glucuronide	[20,33]	[27,29,34]	[29]	[26] (this study)	[28]	-	[29,34]
Apigenin glucuronide	-	-	-	[23]	-	-	-
Luteolin	[20]	[34]	-	[26]	[28]	-	[34]
Luteolin 3′-*O*-glucuronide	[20]	[29,34]	[29]	[26] (this study)	[28]	[22]	[29,34]
Luteolin 4′-*O*-glucuronide	[20]	-	-	[26] (this study)	-	[21]	-
Luteolin 7-*O*-glucuronide	[20]	[27,29,34]	[29]	[23,26] (this study)	[28]	[21,22]	[29,34]
Luteolin 3′,4′-di-*O*-glucuronide	-	[29,34]	[29]	-	-	-	[29,34]
Luteolin 7,3′-di-*O*-glucuronide	[20]	[29,34]	[29]	[23,26] (this study)	[28]	[21,22]	[29,34]
Luteolin 7,4′-di-*O*-glucuronide	[20]	[27,29,34]	[29]	[23]	[28]	-	[29,34]
Luteolin 7,3′,4′-tri-*O*-glucuronide	-	[34]	-	-	-	-	[29,34]
Baicalein 6,7-di-*O*-glucopyranuronoside	[33]	-	-	-	-	-	-
Chrysoeriol 7-*O*-neohesperidoside	[33]	-	-	-	-	-	-

^1^ Old synonyms of *M. polymorpha* L. subspecies [35,36,37,38]: subsp. *polymorpha* (*M. polymorpha* L. f. A α *aquatica* Nees, *M. aquatica* (Nees) Burgeff, *M. polymorpha* var. *aquatica* (Nees) Gottsche); subsp. *ruderalis* (*M. polymorpha* L. f. βγ *domestica* Nees, *M. polymorpha* L. *sensu stricto*, *M. polymorpha* var. *polymorpha* auct., *M. latifolia* Gray); and subsp. *montivagans* (*M. polymorpha* L. f. B *alpestris* Nees, *M. alpestris* (Nees) Burgeff, *M. polymorpha* var. *alpestris* (Nees) Gottsche, Lindenb. and Nees).

The flavones found in our study coincided with some of those previously found in *M. polymorpha* subsp. *ruderalis*, which has been the most experimentally used subspecies, especially in recent years (Table 2). Moreover, four of the six flavones found in our study (including the major one, apigenin 7-*O*-glucuronide) have also been found in all the *M. polymorpha* genotypes analyzed to date, irrespective of the subspecies or accession used. However, the three subspecies of *M. polymorpha* (*polymorpha*, *ruderalis*, and *montivagans*), and even the different accessions of the subsp. *ruderalis*, show some differences between their flavones (Table 2). In addition, nomenclature within the species *M. polymorpha* is somewhat confusing because of historical reasons [35,36,37,38], and there is a current proposal to treat subspecies at species level [39] on the basis of morphology and ecological segregation. Given these facts, and the increasing use of *M. polymorpha* subsp. *ruderalis* as a model organism in physiological, phylogenetic, and molecular studies [19], it is strongly recommended to specify the subspecies and (if pertinent) the accession used in each study, because the results obtained in each subspecies and accession could vary due to genetic reasons. In addition, the environmental or culture conditions would be another source of variability in the phenolic composition of each genotype.

### 3.2. Effects of UV Radiation

The radiation regime strongly influenced SUVACs in *M. polymorpha* subsp. *ruderalis*, interacting with the developmental stage of the samples. The juvenile samples (G and T1 stages) strongly reacted to UV radiation, although the UV levels applied were moderate ([40] see Materials and Methods). In G and T1 plants, the bulk level of SUVACs and the contents of all the individual phenolic compounds (apigenin and luteolin derivatives) showed significantly higher values under UV radiation. This high reactiveness was expected because, in recent studies, the phenolic compounds of *M. polymorpha* have been found to be highly UV-responsive. This has been proven in local wild accessions of subsp. *polymorpha* [27], Tak-1 accession of subsp. *ruderalis* [23,24,25,26] (this study), and local wild accessions of the same subspecies [21,22,28]. The third currently recognized subspecies of *M. polymorpha* (subsp. *montivagans*) has not been tested in this regard yet. In a pioneer study, Markham et al. [20] also found a similar response to ours, although the increase was non-significant and the subspecies and accession of the material used was not unequivocally identified. All these results are in line with the fact that liverworts are, in general, notably UV-responsive, particularly in comparison with mosses [28,41]. In addition, the accumulation of phenolic compounds is the most common response to UV in bryophytes and tracheophytes [18,41,42,43]. This is logical because UV radiation (both UV-B and UV-A) induces the expression of different genes of the flavonoid pathway, in some cases mediated by the UV-B photoreceptor UVR8 [18,21,22,24].

Interestingly, the phenolic compounds showing this consistent response of *M. polymorpha* to UV have always been flavones, specifically apigenin and luteolin derivatives [20,21,22,26,27,28] (this study). Other plants have also shown a similar response of flavones to UV radiation under diverse UV conditions, including tracheophytes [44,45,46,47] and mosses such as *Bryum argenteum* [48] and *Pohlia nutans* [49]. The contribution of flavones to UV tolerance is probably based on their double role as antioxidants and UV screens [31,50], as occurs with other flavonoids (such as flavonols) in other plants [51,52]. Apparently, the early acquisition of flavone biosynthesis by liverworts was an important stress adaptation during land plant evolution [50], given the crucial role of bryophytes as one of the first lineages of embryophytes colonizing land [24,53]. In this sense, the chemical evolution of flavones to other phenolic compounds (such as flavonols and anthocyanins) facilitated plant adaptation to the terrestrial ecosystem [54].

### 3.3. Effects of Developmental Stage

In our study, the effect of UV radiation on the phenolic compounds was clearly modulated by the developmental stage of the liverwort samples, which influenced all the variables measured (except luteolin 7-*O*-glucuronide). When plants were exposed to UV starting from juvenile stages (gemmae or 1-month age thalli), all the SUVACs increased in UV-exposed samples with respect to non-exposed samples. However, when starting from more mature thalli (2-month age), only apigenin derivatives increased and in a more modest manner, whereas the bulk level of SUVACs and luteolin derivatives did not show any difference associated with UV radiation (except luteolin 3′-*O*-glucuronide, which decreased in PAB samples). In addition, although both G and T1 plants accumulated phenolic compounds, the magnitude of the difference between UV-exposed and non-exposed plants was higher in G than in T1 plants for most of the variables measured (the bulk level of SUVACs and most mono-glucuronide derivatives), although not for the di-glucuronide derivatives. Two clear consequences can be derived from these results: (1) the phenolic compounds of *M. polymorpha* subsp. *ruderalis* responded to UV radiation in every developmental stage, but their UV responsiveness decreased with the thallus age; and (2) in UV-exposed plants, different compounds showed different temporal patterns of accumulation: most compounds decreased with thallus age, but di-glucuronide derivatives showed a bell-shaped pattern, with T1 plants showing the highest contents.

There is little information about the influence of the developmental stage on the effects of UV radiation in bryophytes. In the model moss *Physcomitrella* (*Physcomitrium*) *patens*, the growth of the juvenile stages (protonema) was more negatively affected by UV-B radiation than that of the adult gametophores [55], but the phenolic composition of the moss in the different stages was not investigated. In some tracheophytes, as occurred in our study with *Marchantia* thalli, the contents of phenolic compounds increased more strongly in young than old leaves when they were exposed to UV [11,12,13,14]. This would enhance the UV photoprotection of young photosynthetic organs (leaves or thalli), whereas older organs (in our study, T2 thalli) could alternatively be protected through thicker cuticles, or higher contents of dry matter, as occurred in *Arabidopsis* leaves [12]. Moreover, the accumulation of phenolic compounds in young organs would increase their cold and desiccation tolerance [56], as well as their defense against herbivores [57], counteracting in our case their higher softness and attractiveness, derived from their higher WC. However, inverse results (UV radiation increased phenolic compounds in old leaves) were obtained in other tracheophytes [15,16,17], and in *Citrus* the flavonoid content was increased by UV-B radiation in both young and old leaves [58]. Thus, in tracheophytes, the effect of the developmental stage on the induction of phenolic compounds by UV radiation depends on the species considered. In bryophytes, the influence of the species in this respect is still unknown.

Our finding that the temporal patterns of the accumulation of phenolic compounds in UV-exposed plants were compound-specific is supported by results obtained in the leafy liverwort *Jungermannia cordifolia* [4] and in barley [13]. For example, when the liverwort was exposed to UV, two coumarins accumulated in either the young or old segments of the shoot, phaselic acid decreased in the younger parts, feruloylmalic acid accumulated in the older parts, and other compounds showed no clear accumulation pattern along the shoot. One of the reasons explaining this specificity of response to UV radiation, associated with the developmental stage of the plant, is the complexity of the phenolic biosynthesis pathway, which is regulated by many enzymes and genes [21,22,50]. Globally, apigenins were the most UV-responsive individual compounds in our study, increasing irrespectively of the developmental stage. This may be surprising because the antioxidant capacity of apigenins (monohydroxy B-ring-substituted forms) is lower than that of luteolins (dihydroxy B-ring-substituted flavonoids) [59], whereas the ability of both pigments to absorb UV wavelengths would be similar [60]. Thus, it would be expected that luteolins would increase more strongly than apigenins in response to UV radiation. Further studies are needed to establish the specific role played by these compounds in *M. polymorpha*.

Although, as it has been mentioned above, the accumulation of phenolic compounds is the most common response to UV in bryophytes, many studies did not obtain this result, especially in mosses [41]. One of the reasons underlying this fact could be the experimental use of relatively old plants with a low UV responsiveness, as occurred in the T2 thalli in our experiment.

### 3.4. Ordination of Samples by PCA

PCA ordination summarized the findings described above (Figure 3). PC1 represented the response to UV radiation, whereas PC2 represented the developmental stage, ordinating only P samples as a function of their developmental stage. The most UV-responsive plants, showing the highest levels of SUVACs and apigenin and luteolin mono-glucuronides, were G plants of the PAB regime. T1 plants of the PAB regime showed a more modest response, whereas T2 plants were hardly separated from plants of the P regime, which had not been exposed to UV. Thus, as age increased, the responses to UV were weaker. Nevertheless, there was a certain response of apigenin and luteolin di-glucuronides to a combination of age and UV radiation, and the samples best responding to this combination were the intermediate-aged plants that had been exposed to UV (T1 plants of the PAB regime). In particular, it seemed that apigenin mono-glucuronide would represent the typical response to UV (shown by G plants) after a period of exposure of 38 days. Then, apigenin mono-glucuronide would be converted to apigenin di-glucuronide in a more delayed response (shown by T1 plants) over the following month of exposure. Afterwards, phytochemical responses to UV would be more diffuse, probably by senescence and a consequent decrease in metabolic responsiveness and acclimation capacity, although the thalli would become progressively tougher due to continuously decreasing WC, and this could represent a structural protection against excess UV [12]. These characteristics would be exemplified by T2 plants. As for P plants, they would respond only to age (PC2), with lower WC and modestly higher di-glucuronide derivative contents as age increased.

## 4. Materials and Methods

### 4.1. Plant Material and Culture Conditions

Gemmae of a male accession (Takaragaike-1, Tak-1) of the thalloid liverwort *Marchantia polymorpha* L. subsp. *ruderalis* Bischl. and Boissel.-Dub. were obtained from Prof. Takayuki Kohchi (Kyoto University) and cultivated in Petri dishes using ½ Gamborg’s B5 medium. Sixteen gemmae per dish were sown in a regular 4 × 4 grid, and six dishes with gemmae were placed in a growth chamber (Fitoclima 1200, Aralab, Rio de Mouro, Portugal) under 22 °C temperature, 50% relative humidity, and around 65 μmol m^−2^ s^−1^ of photosynthetically active radiation (PAR). After one and two months, two other sets of six dishes were similarly sown and placed under the same conditions, for a total of three sets of six dishes each. At that moment, each set consisted of plants of a different age and developmental stage: two-month thalli (T2), one-month thalli (T1), and just-sown gemmae (G). All the 18 Petri dishes were placed in a growth room at 22 °C, 75% relative humidity, and a 10:14 photoperiod (light:darkness). PAR, UV-A, and UV-B radiations were then provided by, respectively, LED-PAR tubes (LED T8 Tube, AOSZX Brilliant Crystal Co., Shenzhen, China), UV-A lamps (Actinic BL 40W RS, Philips, Amsterdam, Netherlands), and narrowband UV-B lamps (TL40W/01 RS UV-B Narrowband, Philips, Amsterdam, Netherlands).

**Figure 4:**
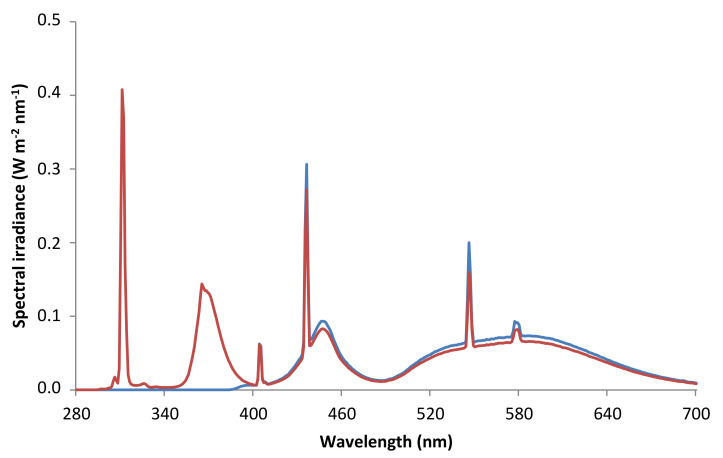
Spectral irradiance (280–700 nm) received by samples of the thalloid liverwort *Marchantia polymorpha* subsp. *ruderalis* (Tak-1 accession) under the two radiation regimes imposed in the experiment: P (only PAR, blue line), and PAB (PAR + UV-A + UV-B, red line).

Different cut-off filters were placed on the Petri dishes to impose two different radiation regimes on the plants (Figure 4):

- P (only PAR), using XT Vitroflex 395 Solarium Incoloro (Polimer Tecnic, Girona, Spain), which cut off UV radiation.
- PAB (PAR + UV-A + UV-B), using Ultraphan 295 (Digefra GmbH, Munich, Germany), which cut off UV-C radiation.

Table 3 shows the radiation conditions imposed on the two regimes used, including the biologically effective UV-B irradiance (UV-BBE) and the biologically effective UV irradiance (UVBE), which were calculated following Caldwell [61], and Flint and Caldwell [62], respectively. The spectral irradiances (Figure 4) were measured using a spectroradiometer (Macam SR9910, Macam Photometrics Ltd., Livingstone, Scotland). UV-A and UV-B irradiances received by the PAB plants were approximately 5% and 90%, respectively, of those recorded in mid-latitudes in summer [40]). The PAB plants received UV-BBE and UVBE daily doses of 6.21 and 6.92 kJ m^−2^, respectively.

Plants were cultivated under these conditions during 38 days. Thus, at the end of the culture period, the respective ages of G, T1 and T2 plants were 38 d, 38 d plus one month, and 38 d plus two months. On the last day of treatment, samples were collected at midday and all the variables described below were measured. Three replicates of each developmental stage and radiation regime were used.

### 4.2. Variables Measured

Water content (WC) was measured as the difference between fresh mass (FM) and dry mass (DM: 60 °C for 24 h), and was expressed by unit of DM.

Methanol-soluble UV-absorbing compounds (SUVACs) were analyzed following Fabón et al. [63]. Frozen apices were ground in a TissueLyser (Qiagen, Hilden, Germany), and then 2 mL of methanol:water:7 M HCl (70:29:1, *v*/*v*/*v*) was added for extraction (24 h at 4 °C in the dark). The extract was centrifuged to precipitate the insoluble fraction, and the bulk levels of SUVACs were measured in the supernatant as the area under the absorbance curve in the intervals 280–315 (AUC280–315), corresponding to the absorbance in the UV-B range, and 280–400 nm (AUC280–400), corresponding to the absorbance in the UV-B plus UV-A ranges, using an Agilent 8453 UV-Visible spectrophotometer (Agilent Technologies, Palo Alto, CA, USA). AUC280–315 and AUC280–400 were expressed per DM unit. Given that both variables rendered similar results, only AUC280–400 will be used in the text.

Individual SUVACs were analyzed in the supernatant by ultra-performance liquid chromatography (UPLC) using a Waters Acquity UPLC system (Waters Corporation, Milford, MA, USA) [28]. Solvents were: A, water/formic acid (0.1%), and B, acetonitrile with 0.1% formic acid. The gradient program employed was: 0–7 min, 99.5–80% A; 7–9 min, 80–50% A; 9–11.7 min, 50–0% A; 11.7–15 min, 0–99.5% A. The UPLC system was coupled to a micrOTOF-QII-ESI-MS/MS high-resolution mass spectrometer (Bruker Daltonics, Bremen, Germany) controlled by the Bruker Daltonics DataAnalysis software. The electrospray source was operated in negative mode. The capillary potential was set to 4 kV; the drying gas temperature was 200 °C and its flow 9 L min^−1^; the nebulizer gas was set to 3.5 bar and 25 °C. Spectra were acquired between *m*/*z* 120 and 1505 in negative mode. Individual SUVACs were identified based on absorption spectra and UPLC-micrOTOF-QII-ESI-MS/MS fragmentation data, and the order of elution reported in Markham et al. [20]. Individual compounds were quantified by integrating peak areas from chromatograms at 324 nm. The standards apigenin 7-*O*-glucuronide (for the same compound), apigenin (for the other apigenin), and luteolin (for luteolins) were used for external calibration curves. Concentrations of the different compounds were quantified as equivalents of their respective standards, and expressed in mg g^−1^ DM. Standards were obtained from Sigma-Aldrich (St. Louis, MO, USA).

### 4.3. Statistical Analysis

Once proved that the data met the assumptions of normality (Shapiro-Wilk’s test) and homoscedasticity (Levene’s test), the global effects of age, radiation regime, and their combination were tested using a two-way analysis of variance (ANOVA), followed by a Tukey’s post-hoc test to compare means for age classes. For each age, differences between the two radiation regimes were tested using Student’s *t*. The samples of the different ages and radiation regimes were ordinated by Principal Components Analysis (PCA) taking into account all the physiological variables measured. All the statistical procedures were performed with SPSS 24.0 for Windows (SPSS Inc., Chicago, IL, USA).

## 5. Conclusions

The effect of UV radiation on the phenolic composition of the liverwort *M. polymorpha* subsp. *ruderalis* (Tak-1 accession) was modulated by the developmental stage of the thallus. The highest responsiveness of phenolic compounds to UV would occur during the first 40 days after gemmae sprouting. In this period, apigenin and luteolin mono-glucuronides, and (to a lesser extent) di-glucuronides, would accumulate, contributing to an increasing bulk level of SUVACs. Then (around 60–70 days after gemmae sprouting), the metabolic responsiveness would decrease as the developmental stage (age) progressed, and a metabolic shift to transform mono-glucuronides into di-glucuronides would take place. This shift would depend on both UV radiation and time. Finally, starting from an age of around 90 days, metabolic responses to UV would be very modest, yet affecting apigenin derivatives (which would still increase) and conserving moderate bulk levels of SUVACs. At this stage, the plant could mainly benefit from the constitutive levels of SUVACs previously accumulated and the still inducible apigenin derivatives. In addition, the UV-protecting metabolic responses would be complemented by structural responses, such as the progressively tougher thallus texture.

The pattern described above should be taken into account in experiments in which the accumulation of UV-inducible compounds is expected, in order to select the adequate thalli age to obtain positive results. In addition, this pattern could explain the relatively low UV-responsiveness shown by *M. polymorpha* in some cases [20], as well as the saturation-type regression between the bulk levels of SUVACs and the accumulated UV dose obtained by Soriano et al. [28].

Given that this is the first study (to our knowledge) on the influence of the developmental stage on the accumulation of phenolic compounds in UV-exposed bryophytes, further research is needed to confirm or reject the hypotheses launched. Finally, given that the radiation conditions used in our study were different from those present in nature, the ecological significance of the results obtained may be limited.

## Figures and Tables

**Table 1:** Overall effects of the radiation regime (P and PAB) and the developmental stage (G, gemmae; T1, one-month thalli; and T2, two-month thalli) on the variables measured in *Marchantia polymorpha* subsp. *ruderalis* Tak-1 samples after 38 days of treatment. Significance levels of *p* for the overall effects of radiation regime (*p*—Rad), developmental stage (*p*—DS), together with the interactions between both factors (*p*—Rad × DS), are shown (two-way ANOVA): ***, *p* < 0.001; ns, non-significant. In the post-hoc Tukey’s test performed to compare developmental stages, different letters (italics) mean significant differences between stages (at least *p* < 0.05), and different intensities of green indicate higher or lower values of each variable following the scale: dark green > medium green > light green. SUVAC, bulk level of methanol-soluble UV-absorbing compounds.

	*p*—Rad	*p*—DS	*p*—Rad × DS	*post-hoc* (DS)		
Water Content	ns	***	ns	**G *a***	T1 *b*	T2 *c*
SUVAC	***	***	***	**G *a***	T1 *b*	T2 *b*
Apigenin 7-*O*-glucuronide	***	***	***	**G *a***	T1 *b*	T2 *c*
Luteolin 3′-*O*-glucuronide	***	***	***	**G *a***	T1 *a*	T2 *b*
Luteolin 4′-*O*-glucuronide	***	***	***	**G *a***	T1 *b*	T2 *c*
Luteolin 7-*O*-glucuronide	***	ns	***	G *a*	**T1 *b***	T2 *a*
Apigenin 7,4′-di-*O*-glucuronide	***	***	***	G *a*	**T1 *b***	T2 *c*
Luteolin 7,3′-di-*O*-glucuronide	***	***	***	G *a*	**T1 *b***	T2 *c*

**Table 3:** Radiation conditions in the two radiation regimes used: P (only photosynthetically active radiation, PAR) and PAB (PAR + UV-A + UV-B). Biologically effective UV-B radiation (UV-B_BE_) and biologically effective UV radiation (UV_BE_) were calculated using the action spectra by Caldwell [61] and Flint and Caldwell [62], respectively.

Radiation	P	PAB
PAR (μmol m^−2^ s^−1^)	67.1	66.5
PAR (W m^−2^)	13.9	13.9
UV-A (W m^−2^)	0.06	3.27
UV-B (W m^−2^)	0.00	1.38
UV-B_BE_ (W m^−2^)	0.00	0.17
UV_BE_ (W m^−2^)	0.00	0.19

## Data Availability

Data supporting reported results can be found in Table S1 (Supplementary Materials).

## Supplementary Materials

The following are available online at https://www.mdpi.com/2223-7747/10/3/473/s1, Table S1: data supporting reported results.
